# Variable ventilation and Jensen's inequality: citation corrections

**DOI:** 10.1186/s13054-016-1335-0

**Published:** 2016-06-17

**Authors:** W. Alan C. Mutch, M. Ruth Graham, John F. Brewster

**Affiliations:** Department of Anesthesia and Perioperative Medicine, University of Manitoba, Winnipeg, Canada; Deparment of Statistics, University of Manitoba, Winnipeg, Canada

We would like to commend Huhle and co-authors [1] for their extensive review of variable ventilation featuring citations from our pioneering work. We would suggest a couple of corrections to the cited articles, however. The authors reference important work by Venegas and colleagues [2], including Figure 4 in their manuscript. This figure explains the convex and concave portions of the sigmoidal pressure-volume curve indicating where variable ventilation has beneficial and detrimental effects, respectively, as suggested by Jensen’s theorem. No mention of Jensen’s ‘theorem’ or ‘inequality’ is discussed in the paper by Venegas et al. This explanation is advanced in a paper by us and not cited (‘Convexity, Jensen’s inequality and benefits of noisy mechanical ventilation’ [3]). The data supplement in our paper gives a comprehensive mathematical explanation for conditions where non-linear systems can and cannot benefit from the addition of noise. Mechanical ventilation is but one example where noisy life support systems may provide benefit to patients.
