# Supplementary material for: Variable ventilation and Jensen's inequality: citation corrections
Source: Crit Care. 2016 Jun 17;20:190. doi: 10.1186/s13054-016-1335-0 (PMC4911688; doi:10.1186/s13054-016-1335-0)
Supplement: Related texts

# Reply: Authors’ response

**Authors:** Robert Huhle, Paolo Pelosi, Marcelo Gama de Abreu

We thank Drs. Mutch, Graham, and Brewster for their interest in our review article [1], and their invaluable contribution to the field of variable ventilation. We are aware of the outstanding publication that the authors of the letter missed in our article [3].

We would like to briefly explain the reasons for not including it in our review [1].

The mechanism usually referred to as “Jensen’s inequality” [3] is in fact the mathematical theorem forming the basis of stochastic resonance [4] on the convex relationship between volume and pressure of the respiratory system. Thus, while the formal more detailed description of the “Jensen’s inequality” [3] contributed to understand one of the mechanisms of variable ventilation, its basis was described years before [4]. We are aware of the fact that the Venega’s equation [2] does not describe the “Jensen’s inequality” theorem. Rather, the equation characterizes mathematically the sigmoidal relationship between pressure and volume of the respiratory system. Thus, we combined the primary concept of stochastic resonance [4] with a concave pressure-volume relationship [2] to also call attention to possible detrimental effects of variable ventilation, which were not mentioned before [3].

Most importantly, we must recognize the effort of different research groups in experimental settings showing beneficial effects of variable ventilation on respiratory function and reduction in ventilator-associated lung injury. We hope that future randomized clinical studies will determine the effects of variable ventilation on clinical outcome.

In view of these facts, and due to the journal’s restrictions regarding the number of references, we included original concepts and relevant clinical and physiological studies only, including some from Dr. Mutch’s group.
